# How Close is too Close? The Effect of a Non-Lethal Electric Shark Deterrent on White Shark Behaviour

**DOI:** 10.1371/journal.pone.0157717

**Published:** 2016-07-01

**Authors:** Ryan M. Kempster, Channing A. Egeberg, Nathan S. Hart, Laura Ryan, Lucille Chapuis, Caroline C. Kerr, Carl Schmidt, Charlie Huveneers, Enrico Gennari, Kara E. Yopak, Jessica J. Meeuwig, Shaun P. Collin

**Affiliations:** 1 The Oceans Institute and the School of Animal Biology, The University of Western Australia, Crawley, Western Australia, Australia; 2 Department of Biological Sciences, Macquarie University, North Ryde, New South Wales, Australia; 3 School of Biological Sciences, Flinders University, Bedford Park, South Australia, Australia; 4 Oceans Research, Mossel Bay, South Africa; 5 South African Institute for Aquatic Biodiversity, Private Bag 1015, Grahamstown, South Africa; 6 The Oceans Institute and the Centre for Marine Futures, School of Animal Biology, The University of Western Australia, Crawley, Western Australia, Australia; Department of Agriculture and Water Resources, AUSTRALIA

## Abstract

Sharks play a vital role in the health of marine ecosystems, but the potential threat that sharks pose to humans is a reminder of our vulnerability when entering the ocean. Personal shark deterrents are being marketed as the solution to mitigate the threat that sharks pose. However, the effectiveness claims of many personal deterrents are based on our knowledge of shark sensory biology rather than robust testing of the devices themselves, as most have not been subjected to independent scientific studies. Therefore, there is a clear need for thorough testing of commercially available shark deterrents to provide the public with recommendations of their effectiveness. Using a modified stereo-camera system, we quantified behavioural interactions between white sharks (*Carcharodon carcharias*) and a baited target in the presence of a commercially available, personal electric shark deterrent (Shark Shield Freedom7^™^). The stereo-camera system enabled an accurate assessment of the behavioural responses of *C*. *carcharias* when encountering a non-lethal electric field many times stronger than what they would naturally experience. Upon their first observed encounter, all *C*. *carcharias* were repelled at a mean (± std. error) proximity of 131 (± 10.3) cm, which corresponded to a mean voltage gradient of 9.7 (± 0.9) V/m. With each subsequent encounter, their proximity decreased by an average of 11.6 cm, which corresponded to an increase in tolerance to the electric field by an average of 2.6 (± 0.5) V/m per encounter. Despite the increase in tolerance, sharks continued to be deterred from interacting for the duration of each trial when in the presence of an active Shark Shield^™^. Furthermore, the findings provide no support to the theory that electric deterrents attract sharks. The results of this study provide quantitative evidence of the effectiveness of a non-lethal electric shark deterrent, its influence on the behaviour of *C*. *carcharias*, and an accurate method for testing other shark deterrent technologies.

## Introduction

Although relatively rare, shark bite incidents draw a high level of interest from both the public and the media [1], as they often result in serious consequences for those involved. Globally, the number of negative shark encounters has increased, which has largely been attributed to population increases and more people entering the ocean [2]. Nevertheless, the threat that sharks pose to ocean users has led to the adoption of a range of shark control programs around the world that often involve the removal of sharks to reduce risk [3–6]. However, these programs are in conflict with the important ecological role that predatory sharks play in ocean ecosystems [7,8]. Since these control programs do not discriminate by species or size, they place increased pressure on non-target and potentially vulnerable species [9–12]. The effects of removing sharks from our oceans, although complex, can be ecologically and economically damaging [8,13–17]. There is, therefore, a clear need for alternative non-lethal shark mitigation solutions that will allow humans and sharks to safely co-exist.

Current research suggests that there are a variety of methods that could be used to deter sharks from an area based purely on manipulation of their sensory behaviours [18–20]. Shark deterrents offer the potential of a non-lethal solution to protect individuals from negative interactions with sharks, while reducing the need for lethal shark control strategies. There are a number of shark deterrent technologies available to the public, but little scientifically robust testing has been conducted on their effectiveness [21,22]. However, one promising form of sensory deterrent, which utilises electric fields in an attempt to over-stimulate a sharks’ electrosensory system, is gaining positive attention [21–25], and, furthermore, has been shown to have minimal effect on other species that do not possess an electrosensory system [26].

The electrosensory organs of sharks, known as the ampullae of Lorenzini, detect minute electric field gradients (≤1 nV/cm) via an array of small pore openings on the surface of the head [27]. The electrosensory system is known to mediate the passive detection of bioelectric stimuli produced by potential prey [27–30], predators [31,32], and conspecifics [32,33]. This sensory modality is, therefore, used by sharks for both attraction and avoidance, which might lead to potential applications for use in non-lethal electric deterrents.

An innate avoidance of electric fields by sharks was first recognised in 1935 when it was shown that blindfolded small-spotted catsharks (*Scyliorhinus canicula*) would react to the presence of a rusty steel wire when brought in close contact with the head [34]. The galvanic currents generated on the wire’s surface were large enough to be detected by the shark’s electrosensory system, resulting in a behavioural response. Subsequent research demonstrated that sharks can be attracted to electrical fields [35,36], but they can also be repelled when an electric stimulus differs in frequency or strength from the bioelectric fields produced by their prey [31].

Despite much attention being focused on the ability of sharks to detect weak electric field gradients [28,29,37–42], little research has focused on the deterrent threshold of a sharks’ electrosensory system [43–45]. Smith [45] showed that juvenile dusky sharks (*Carcharhinus obscurus*) and other species of sharks would not cross an electric field when field gradients reached 7–10 V/m. Further investigation, which included behavioural tests on bull sharks (*Carcharhinus leucas*), revealed a deterrent threshold as low as 3 V/m [25]. Marcotte and Lowe [43] recorded slightly higher deterrent thresholds of 18.5 V/m and 9.6 V/m for scalloped hammerheads (*Sphyrna lewini*) and leopard sharks (*Triakis semifasciata*), respectively. However, the higher tolerance observed by Marcotte and Lowe [43] was likely due to forced acclimation, as sharks could not completely leave the testing arena. Nevertheless, these studies highlight the potential species-specific variability in deterrent threshold, which will likely be an important consideration in the development of an electric deterrent.

Electric deterrent technology was advanced by the KwaZulu-Natal Sharks Board with the development of the personal deterrent known as the SharkPOD^™^ (Protective Oceanic Device) [22], which was later licensed to an Australian company (Shark Shield^™^) and is currently available for sale to the public under the same name. The Shark Shield^™^ (including its former version, the SharkPOD^™^) is the only electric deterrent to date to have undergone any form of robust scientific scrutiny that has been reported in peer-reviewed literature [21,22,26].

In previous field tests, the Shark Shield^™^ has been shown to increase the time it takes white sharks (*Carcharodon carcharias*) to interact with bait positioned 2–3 m away from the device, but did not prevent interactions altogether [21]. In contrast, when the device was positioned below a seal decoy at the surface, it was shown to significantly decrease the number of breaches and surface interactions [21]. These findings suggest that the Shark Shield^™^ is most effective at close proximity (≤2 m), but it is still unclear what exactly the effective range is and how this relates to the electric field gradient experienced by *C*. *carcharias*.

In the present study, we aimed to determine the effective deterrent radius of the Shark Shield^™^ over time by measuring the closest distance that ‘naive’ and ‘experienced’ *C*. *carcharias* would approach a bait protected by the active device, compared to a visually-identical, but electrically inactive, control. In addition, by measuring the electric field gradient produced by the Shark Shield^™^, this study aimed to determine the strength of the electric field gradient that elicits a deterrent response by *C*. *carcharias*. This will help to guide the development of future electric deterrents, and also provide a basis for assessing the potential effectiveness of other electric deterrents on the market.

## Materials and Methods

### Ethics and Permit Statement

This project was approved by The University of Western Australia Animal Ethics Committee (Permit No. RA/3/100/1193), and by the South African Department of Environmental Affairs: Biodiversity and Coastal Research, Oceans and Coasts Branch (Permit No. RES2014/91). All work was carried out in strict accordance with the guidelines of the Australian Code of Practice for the Care and Use of Animals for Scientific Purposes (8th Edition 2013).

### Study Site

**Fig 1:**
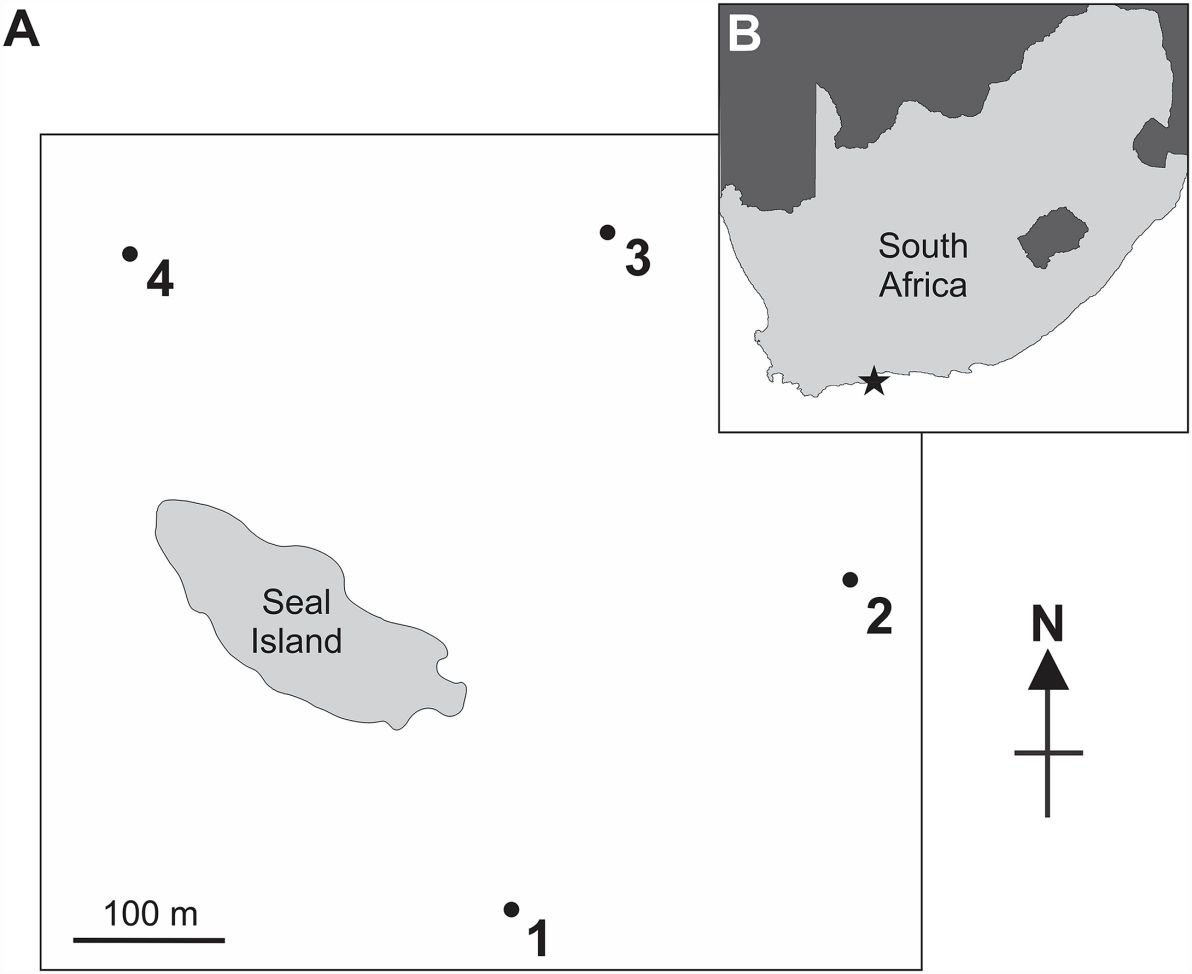
Map of Seal Island (A) in Mossel Bay, South Africa (B), highlighting the specific location of testing sites around the island (A1-4). Testing site locations are not exact, but, instead, mark the approximate area that trials were concurrently conducted.

Experiments were conducted on consecutive days in July 2014 off of Seal Island, Mossel Bay, in the Western Cape region of South Africa (one of nine provinces) (Fig 1). This site was chosen due to its calm conditions and the large population of pinnipeds that frequent Seal Island, which has resulted in a reliably high abundance of *C*. *carcharias* throughout the year [46]. Testing was conducted simultaneously at four locations on the eastern side of the island (Fig 1A) and repeated four times each day between 8 am and 4 pm.

### Remote Monitoring Research Apparatus (ReMoRA)

**Fig 2:**
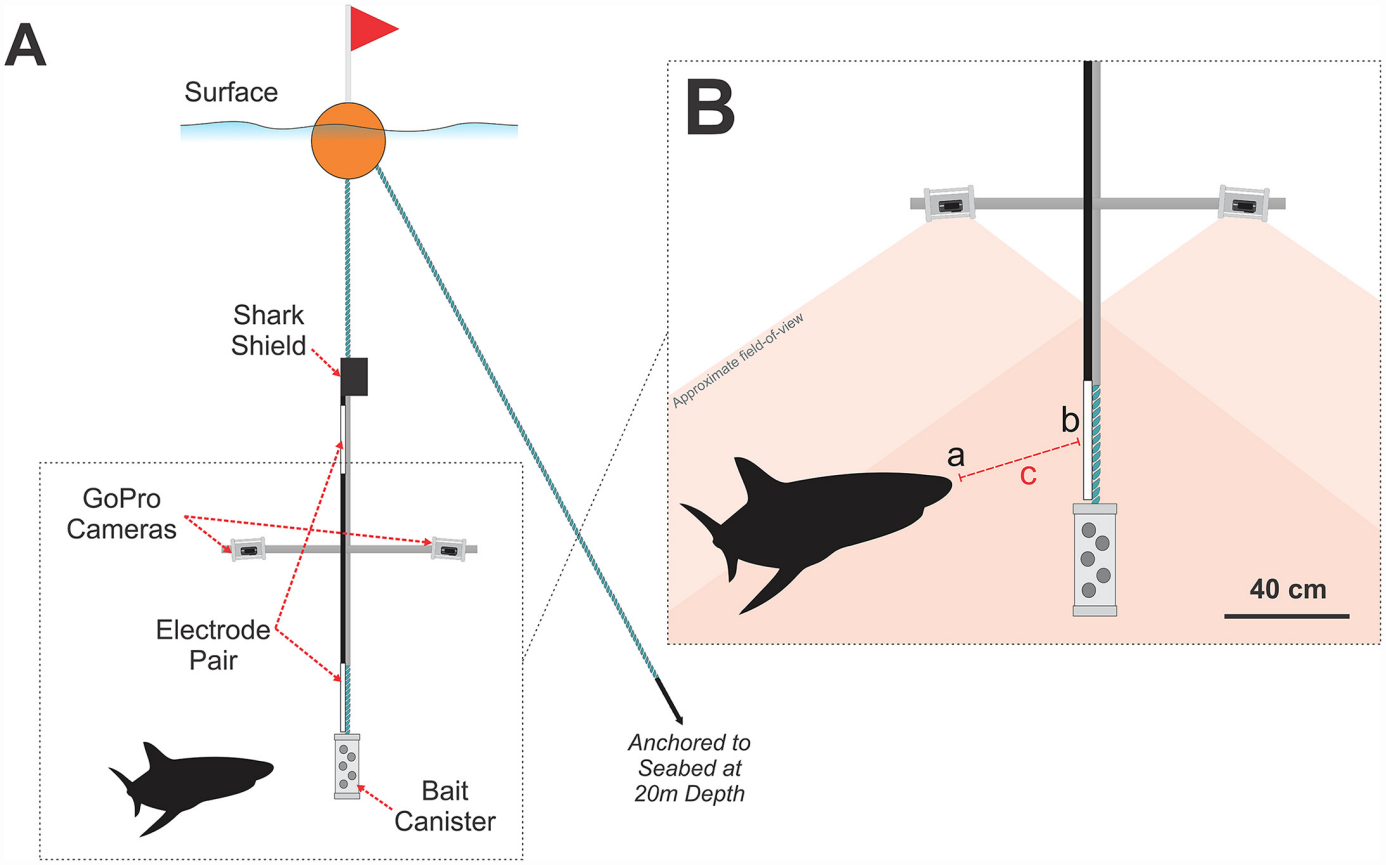
Diagram of a Remote Monitoring Research Apparatus (ReMoRA). (A) Shows the ReMoRA in its deployed configuration with downward facing cameras. (B) Shows the measurements recorded to calculate proximity of *C*. *carcharias* to the visible Shark Shield^™^ electrode. Using Event Measure software, the closest part of a shark’s head to the electrode is marked via the left and right video clips (a), followed by the centre of the Shark Shield^™^ electrode (b). Event Measure compares the length and angle of the lines drawn in the left and right synchronised/calibrated video clips (c) to accurately calculate the closest observable proximity of the shark in three-dimensional space, taking into account both the vertical and horizontal axis. For clarity, the electrodes of the Shark Shield^™^ are displayed in white to highlight their position.

Stereo-video recordings were made using a modified Baited Remote Underwater Video System (BRUVS) called a Remote Monitoring Research Apparatus (ReMoRA) (Fig 2). Stereo-BRUVS have been used extensively to characterise fish assemblages and allow for the recording of events at precise distances [47,48]. The ReMoRA comprised two downward facing GoPro Hero 3^™^ high-definition cameras (in waterproof housings) that were synchronised at the start of each deployment using a clapperboard. The GoPro^™^ cameras recorded at an equivalent fixed focal length of 21 mm, 1080p resolution, 30 frames per second, and were set to a ‘Medium’ field-of-view (horizontal: 94.4°; vertical: 55°). The cameras were positioned 0.7 m apart on a horizontal aluminium square bar, affixed perpendicularly to a vertical stainless-steel pole (Fig 2). GoPro cameras were chosen due to their low cost, and ability to generate accurate length measurements from stereo video footage [48]. The cameras were inwardly converged at 8 degrees to gain a maximum field of view and to allow for three-dimensional calibrations used for fish length measurements [48,49] (Fig 2B). A metal frame was used to ensure that the stereo cameras could not move during deployments, which was essential to maintain stereo calibration of the cameras and ensure the accuracy of proximity measurements. Although metal may theoretically produce a small current, it would not have affected the findings of this study, as the same equipment was used for both the control and active treatments. Furthermore, divers using a Shark Shield would use the device in close proximity to a metal dive tank, and so any potential effect of the metal frame in this investigation likely reflects that of a real use scenario. Nevertheless, it is important to acknowledge that the metal frame has the capacity to conduct electric fields, but we do not believe that it had any significant impact in this investigation. A PVC container (15 cm X 40 cm), holding approximately 0.5 kg of sardines and locally sourced fish heads, was suspended 1 m in front of the cameras to act as a controlled attractant.

### Electric Shark Deterrent

The source of the electric deterrent in this study was the commercially available Shark Shield Freedom 7^™^ (from here on referred to simply as Shark Shield^™^). The Shark Shield^™^ is a portable electronic device that emits an electromagnetic field and is used by recreational water users to repel sharks. The device consists of an electronic control unit, typically attached to the ankle of the user, with a 2.2 m long trailing antenna. The antenna contains two elongated electrode plates separated at its ends (approximately 2 m apart). When the device is turned on and submerged in seawater, the electric circuit is completed, which results in the generation of an electric field thought to be repellent to sharks.

### Experimental Design

ReMoRAs were deployed with either an inactive Shark Shield^™^ (control treatment) or an active Shark Shield^™^ (active treatment). Each rig was suspended from the surface via a large float, with the bait positioned at approximately 4 m depth (1 m below the cameras), and anchored to the seabed at approximately 20 m depth (Fig 2). Four ReMoRAs were deployed simultaneously across four locations on the eastern side of Seal Island (two control and two active), which were each separated by at least 300 m (Fig 1A). After each ReMoRA was deployed, the vessel moved to the other side of the island to avoid interference with the experiment. Once deployed, each ReMoRA (active and control) recorded continuously for 90 minutes to complete one trial. Each ReMoRA was retrieved and redeployed at a different site (after replacing camera batteries, SD cards and bait), rotating randomly between all four sites throughout a day’s testing. Potential temporal and spatial influence was avoided by deploying control and active treatments evenly between locations and at the same period of time each day.

Individual sharks were identified from distinct markings, scars, and fin shapes, using a catalogue of known individuals provided by local researchers at Oceans Research (www.oceans-research.com). An encounter was recorded whenever any part of a shark (head, fin, or body) appeared on the ReMoRA’s video footage (approximately ≤3 m from the bait, depending on the visibility of the water at each location). Furthermore, an interaction was recorded when any part of a shark (head, fin, or body) touched the bait. Accurate assessments of sex were not possible, but based on local knowledge and previous research [50], the population around Seal Island is thought to be made up of predominantly females. Furthermore, due to low visibility, and the large size of sharks being encountered, total length could not always be measured from the ReMoRA stereo-video footage, as the whole shark wasn’t always in the field-of-view at any one time. Nevertheless, based on information from local researchers, all sharks included in this investigation were considered to be sub-adults between 2 and 4 m in total length (E. Gennari, unpublished data).

### Video Calibration

The program CAL^™^ (SeaGIS Pty. Ltd.) was used to calibrate the stereo ReMoRAs using a standard calibration cube [51] before and after completion of the field work in order to ensure accurate proximity measurements were recorded from the video footage collected. The calibration cube was black in colour and had a series of white circular targets on its surface. The targets provided high contrast, unambiguous points, which allowed for a simultaneous, self-calibration of both cameras. Screenshots were taken of the cube as it was rotated through a series of orientations. These screenshots were then used in the CAL program to calibrate the two cameras, which produced a calibration file unique to each ReMoRA and its associated cameras. This calibration file was then uploaded to the program EventMeasure^™^ (SeaGIS Pty. Ltd.) to facilitate the accurate recording of distance/proximity measurements. The whole calibration procedure is described in detail by Harvey and Shortis [49]. Xilisoft^™^ video conversion software (Xilisoft Corporation^™^) was used to merge and convert MP4 GoPro^™^ footage to AVI format to facilitate image analysis using the EventMeasure^™^ program.

### Video Analysis

**Fig 3:**
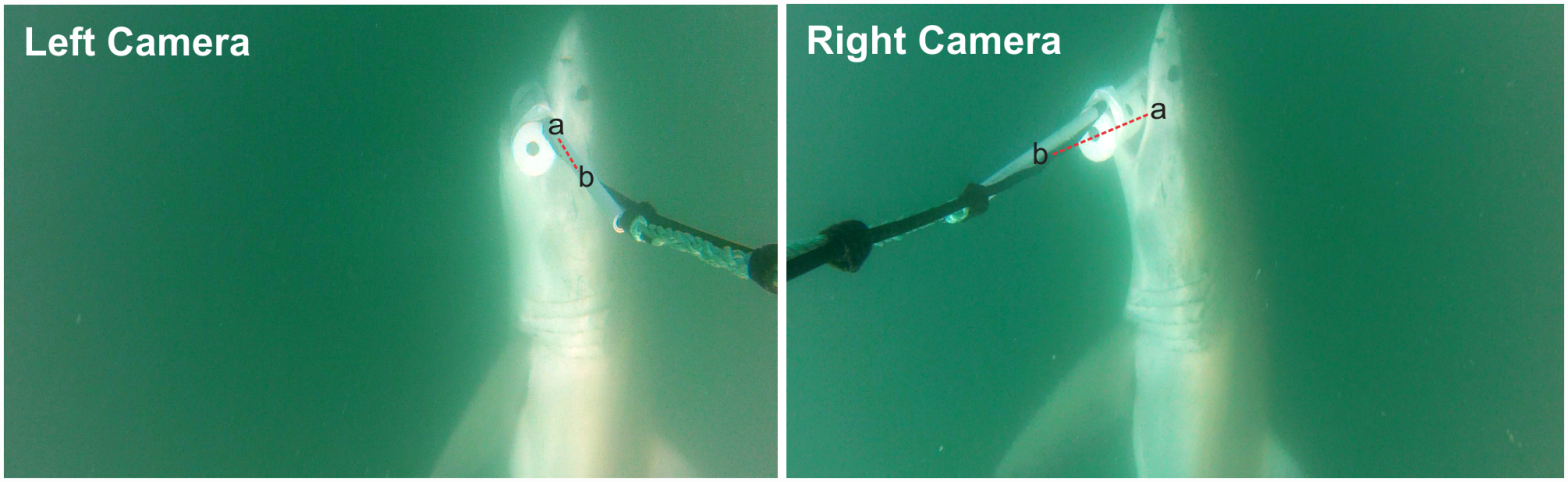
Synchronised video screenshots from the left and right cameras of a ReMoRA, which show *C*. *carcharias* interacting with the bait container during a control trial. The screenshots shown are three-dimensional, so for reference to size see Fig 2B. Using Event Measure software, the distance between a shark’s head (a) and the centre of the Shark Shield^™^ electrode (b) is calculated by comparing the length and angle of lines drawn between these two points on the left and right synchronised/calibrated video clips. The proximity of the shark in the screenshots displayed is 40 cm.

EventMeasure^™^ was used to identify and count the number of individuals, estimate individual lengths (where possible), measure time spent in the area, and quantify minimum distance (proximity) to the deterrent during encounters. The software allowed the synchronisation of calibrated stereo-video footage to facilitate the accurate recording of distance and size measurements in three-dimensional space. Time spent in the area was measured between a shark’s first and last appearance within the field-of-view of the cameras. Proximity measurements were obtained using standardised methods for recording fish lengths, as described in the literature [47–49,52–56], but rather than measuring total length, the distance between the closest part of a shark’s head and the center of the Shark Shield’s^™^ visible electrode was recorded instead (Figs 2 and 3). If poor visibility restricted the observer’s ability to accurately place proximity markers in a specific area of the shark’s head then a more distinct landmark was used instead, such as the eye, nostril or mouth. To ensure consistency, and remove individual bias, a single observer recorded all measurements, which were checked and corroborated by a second observer. A single proximity measurement was calculated for each encounter and defined as the closest observable distance a shark approached during an encounter with the control and active treatment, regardless of whether they interacted with the treatment or not. Therefore, even when a shark interacted by biting a bait canister, their closest proximity to the center of the electrode was still calculated. This allowed for the calculation of the highest electric field strength that a shark experienced during each encounter with an active Shark Shield^™^ (Figs 2 and 3).

### Electric Field Gradient

**Fig 4:**
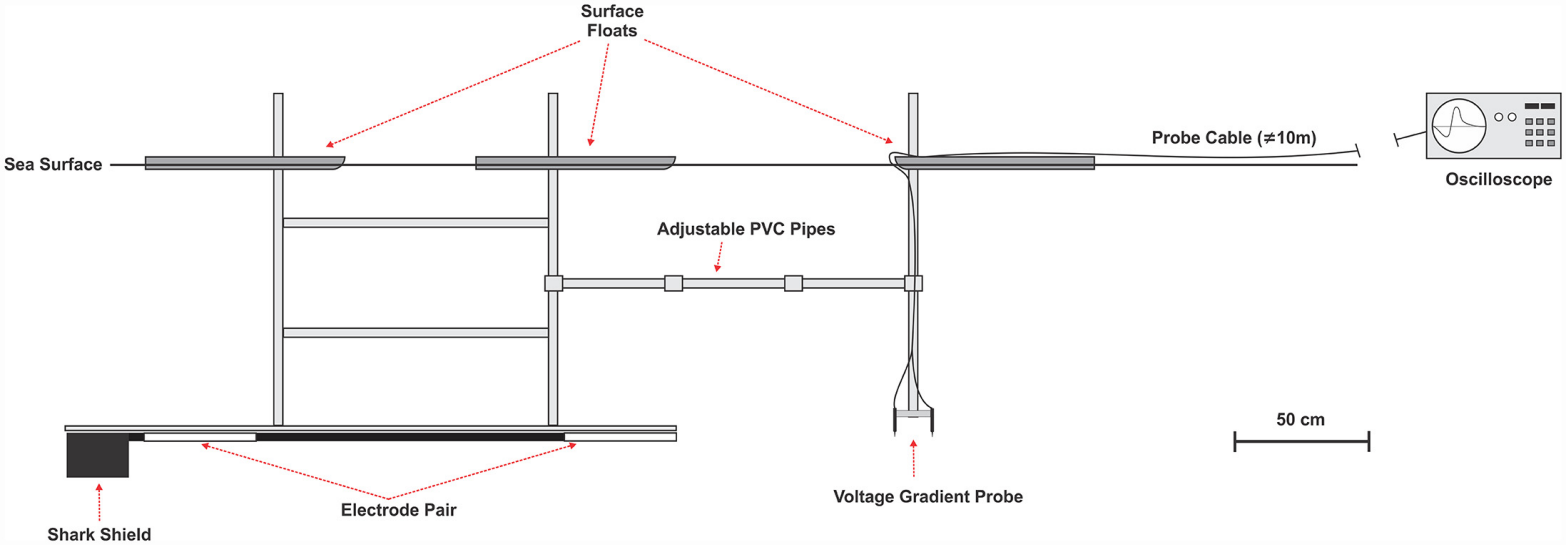
Schematic of the equipment used to measure the voltage gradient of the Shark Shield^™^. For clarity, the electrodes of the Shark Shield^™^ are displayed in white to highlight their position.

To estimate the electric field gradient that a shark experiences during each encounter, a voltage gradient probe was constructed and connected to an oscilloscope to record the electric field gradient at set distances, and angles, relative to an active Shark Shield^**™**^ (Fig 4). The probe consisted of two electrodes separated by 10 cm (a separation distance of 10 cm was necessary to detect a change in voltage gradient over the background noise while maintaining a high enough resolution to accurately determine changes in the electric field gradient with increasing distance). Measurements were recorded at 50 cm intervals proximal to an active electrode in both perpendicular and parallel planes, to determine the effect of the probe’s angel (relative to the electrode) on the field strength recorded. These measurements were then used to plot a curve to estimate the voltage gradient decline with increasing distance. Measurements were recorded in a sheltered bay with a bottom depth of 4 m, at a temperature and salinity consistent with Mossel Bay (15°C; 37 ppt). Due to the shallow depth, the Shark Shield^**™**^ was positioned horizontally to allow the probe to be positioned at distances greater than the vertical depth would have allowed (Fig 4). The shallow depth was also necessary to allow the probe to be accurately positioned by an experimenter and to minimise wave disturbance. However, the proximity of the Shark Shield^™^ to the seabed and the surface is likely to have an effect on the distribution of the electric field. Furthermore, for logistical reasons, the voltage gradient of the Shark Shield^**™**^ was measured by itself, without being attached to a ReMoRA. Therefore, electric field measurements presented in this study should only be used as an estimate and not absolute, as they are likely to vary depending on the conditions in which the device is used. Finally, an inactive Shark Shield^™^ was also measured, to confirm that no voltage gradient (above background noise) was produced when the device was turned off.

### Data Analysis

All encounters of *C*. *carcharias* with a ReMoRA (appearance on the stereo-camera video footage) were classified at three levels of interaction. If a shark passed by (within the field of view of the cameras) without interacting, then it was categorized as type 0 interaction. If a shark touched the bait with any part of its body (other than its mouth), then it was categorized as a type 1 interaction. Finally, if a shark bit the bait, its behaviour was categorised as a type 2 interaction. No individual sharks were identified as appearing in multiple trials; although, we cannot be absolutely certain that this did not occur, as identification was difficult for some encounters. Nevertheless, for statistical purposes, data from different trials were not considered to reflect repeated measures on individual animals. Where relevant to do so, statistical tests were weighted by encounter number or shark ID to detect any affect that individual sharks and/or number of encounters had on each treatment (control and active). All statistical tests were performed using the statistics software Minitab^™^ (Minitab Inc.), and, unless otherwise stated, data are presented as mean ± std. error throughout.

## Results

### Interactions

A total of 44 trials were conducted, of which 22 were deployed with an inactive (control) Shark Shield^™^ and 22 deployed with an active Shark Shield^™^, which resulted in 322 encounters (279 control; 43 active) from 41 *C*. *carcharias* individuals. Sharks were recorded in 68% of the control trials, whereas only 27% of active trials had sharks present (p ≤ 0.05; Table 1: #1). Furthermore, of those individuals that entered the camera’s field-of-view, 83% more of them interacted (includes both type 1 and type 2 interactions) (p ≤ 0.001; Table 1: #2) during control trials (94%) than during active trials (11%). Although, it should be noted that only a single *C*. *carcharias* individual interacted during all active trials, and so the proportion of individuals interacting is likely to appear inflated. Even when only type 2 interactions were considered, there were still significantly more interactions (p ≤ 0.001; Table 1: #3) during control trials (81%) than during active trials (11%). As there were no type 1 interactions during the active trials (see Table 1), and there was no significant difference between the proportion of type 2 and type 1+2 interactions during the control trials (One Sample Proportion test: z = -1.54, p = 0.247), from this point on in the analyses all type 1 and 2 interactions were grouped together.

**Table 1:** Comparison of the behavioural response of *C*. *carcharias* when encountering an inactive (control) or active Shark Shield^™^. For more detailed data, see S1 Table. Justification for the statistical tests used is provided below.

		Control		Active				
Test #	Description (Control vs. Active)	N	(Mean ± Standard Error)	N	(Mean ± Standard Error)	Statistical Test	Test Result	Probability
1	Proportion of deployments with sharks present	22	0.68 ± 0.10	22	0.27 ± 0.10	Two Sample Proportion Test	Z = 2.98	*p ≤ 0*.050*
2	Proportion of sharks interacting	32	0.94 ± 0.04	9	0.11 ± 0.11	Two Sample Proportion Test	Z = 7.30	*p ≤ 0*.001*
3	Proportion of sharks interacting (type 2 only)	32	0.81 ± 0.07	9	0.11 ± 0.11	Two Sample Proportion Test	Z = 5.59	*p ≤ 0*.001*
4	Proportion of sharks interacting (first encounter only)	32	0.59 ± 0.09	9	0.00 ± 0.00	Two Sample Proportion Test	Z = 6.84	*p ≤ 0*.001*
5	No. of encounters/shark	32	8.03 ± 1.02	9	4.44 ± 1.04	Two Sample t-Test^c^^,^^f^	T_16_ = 2.06	p = 0.056
6	No. of interactions/shark	32	6.40 ± 0.84	9	0.22 ± 0.22	Mann-Whitney U Test^d^^,^^f^	W = 57.5	*p ≤ 0*.001*
7	Arrival time of first shark on screen/trial	15	43:06 ± 07:04 mins	6	52:12 ± 17:02 mins	Two Sample t-Test^a^^,^^f^	T_6_ = -0.46	p = 0.664
8	Time taken to first interaction/shark	29	00:11 ± 00:04 mins	1	01:18 ± n/a mins	n/a	n/a	n/a
9	Total time in area/shark	32	01:42 ± 00:16 mins	9	00:58 ± 00:16 mins	Two Sample t-Test^c^^,^^f^	T_14_ = 1.40	p = 0.185
10	Time between encounters/shark	29	00:14 ± 00:01 mins	6	00:19 ± 00:03 mins	Two Sample t-Test^b^^,^^f^	T_7_ = -1.82	p = 0.112
11	Time between encounters/encounter number	8	00:14 ± 00:00 mins	8	00:16 ± 00:02 mins	Paired t-Test^b^^,^^e^	T = -1.13	p = 0.294
12	Proximity (first encounter only)	25	38.10 ± 4.90 cm	6	131.30 ± 10.30 cm	Two Sample t-Test^b^^,^^f^	T_28_ = -8.43	*p ≤ 0*.001*
13	Proximity/shark (all encounters)	29	26.20 ± 2.33 cm	8	98.90 ± 14.80 cm	Two Sample t-Test^b^^,^^f^	T_11_ = -6.93	*p ≤ 0*.001*
14	Proximity/encounter (all sharks)	7	24.01 ± 2.70 cm	7	81.80 ± 11.50 cm	Paired t-Test^b^^,^^e^	T = -10.31	*p ≤ 0*.001*

* Denotes a significant result.

Test justification:

^(a)^ Normal distribution and equal variance;

^(b)^ Normal distribution and equal variance with Log10 transformation;

^(c)^ Normal distribution and equal variance with SqRoot transformation;

^(d)^ Non-normal distribution even after transformation;

^(e)^ Data paired by encounter;

^(f)^ Data unpaired.

Upon their first encounter (appearance within the camera’s field-of-view) with a ReMoRA, 59% of *C*. *carcharias* individuals interacted with the bait during control trials, whereas no sharks interacted on their first encounter during an active trial (p ≤ 0.001; Table 1: #4). On average, *C*. *carcharias* individuals encountered a ReMoRA 8 times during a control trial and 4 times during an active trial, with a statistically borderline difference observed (p = 0.056; Table 1: #5). During control trials, individual sharks interacted with the bait approximately 6 times out of every 8 encounters, whereas, during active trials, less than 1 out of every 4 would result in an interaction (p ≤ 0.001; Table 1: #6).

### Time Taken to Arrive and Interact

The average time taken for *C*. *carcharias* to first arrive did not differ significantly between the control (43 ± 7 mins) and active (52 ± 17 mins) trials (p = 0.664; Table 1: #7). After first arrival, during control trials, *C*. *carcharias* took an average of 11 (± 4) s to interact with the bait canister (Table 1: #8). Sharks continued to encounter the ReMoRA for an average of 1:42 (± 0:16) mins, after which they would not be seen again during that same trial (Table 1: #9). In contrast, the single *C*. *carcharias* individual that interacted during the active trial took 1:18 mins to interact after arriving (Table 1: #8). Of the individuals that were observed during active trials, they would continue to reappear in the camera’s field-of-view for an average of 58 (± 16) s, which was not significantly different from the total time that sharks appeared in the control trials (p = 0.185; Table 1: #9). The time taken for *C*. *carcharias* individuals to reappear in the field-of-view, following a previous encounter, occurred over a short time frame (14–19 s between encounters) with no significant time difference observed between encounters during active or control trials (all p ≥ 0.05; Table 1: #10 and #11).

### Proximity

**Fig 5:**
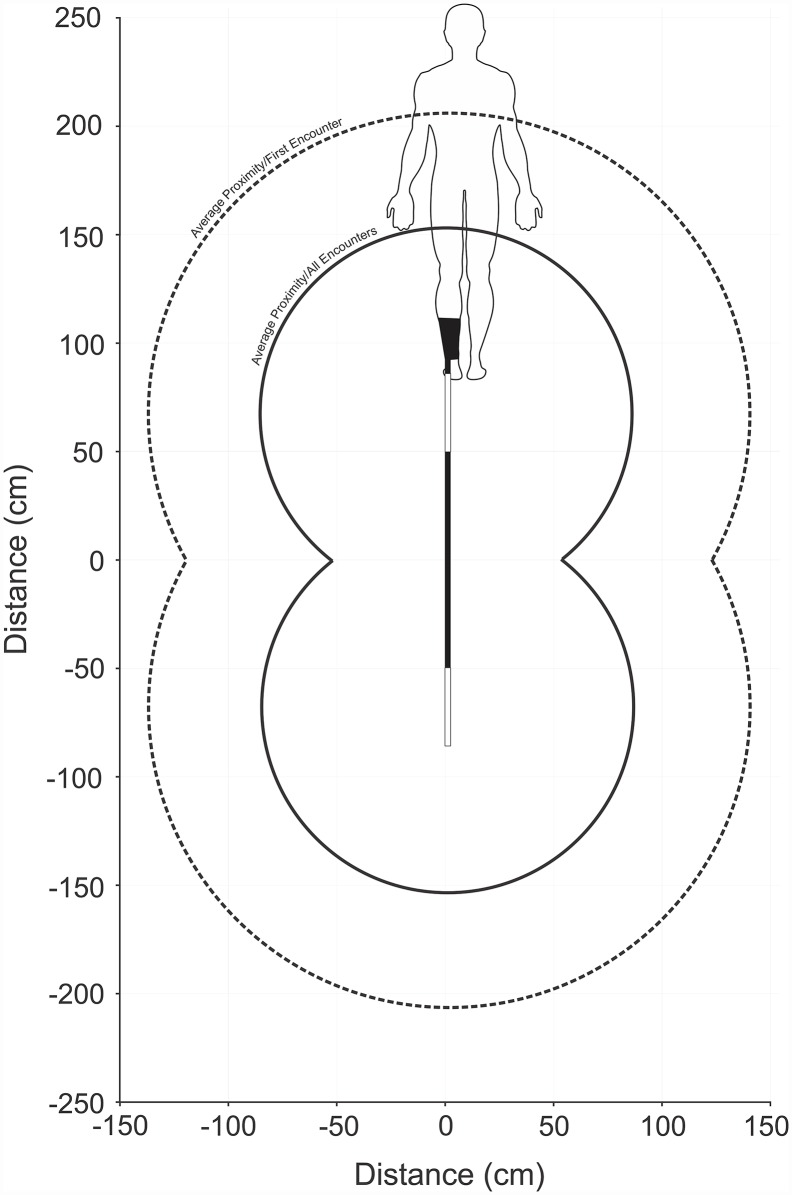
Schematic representation of a user wearing a Shark Shield^™^ with the estimated average deterrent thresholds of *C*. *carcharias* overlaid. The dashed line depicts the average proximity of a shark’s first encounter (131 cm [9.7 V/m]); and the solid line depicts the average proximity of all encounters (82 cm [15.7 V/m]).

The average proximity of the first encounter of *C*. *carcharias* individuals during control trials was approximately 38 (± 5) cm from the inactive Shark Shield^™^ (Table 1: #12). In contrast, during active trials, sharks approached only as close as 131 (± 10) cm from the active Shark Shield^™^ (Fig 5), which was significantly further away than during control trials (p ≤ 0.001; Table 1: #12). When considering all encounters, and weighting for the effect of individuals, the average proximity to the Shark Shield^™^ differed significantly between the control (26 ± 2 cm) and active (99 ± 15 cm) trials (p ≤ 0.001; Table 1: #13). Similarly, when considering all encounters, and weighting for the effect of the encounter number, the average proximity still differed significantly between control (24 ± 3 cm) and active (82 ± 12 cm) trials (p ≤ 0.001; Table 1: #14).

### Habituation

**Fig 6:**
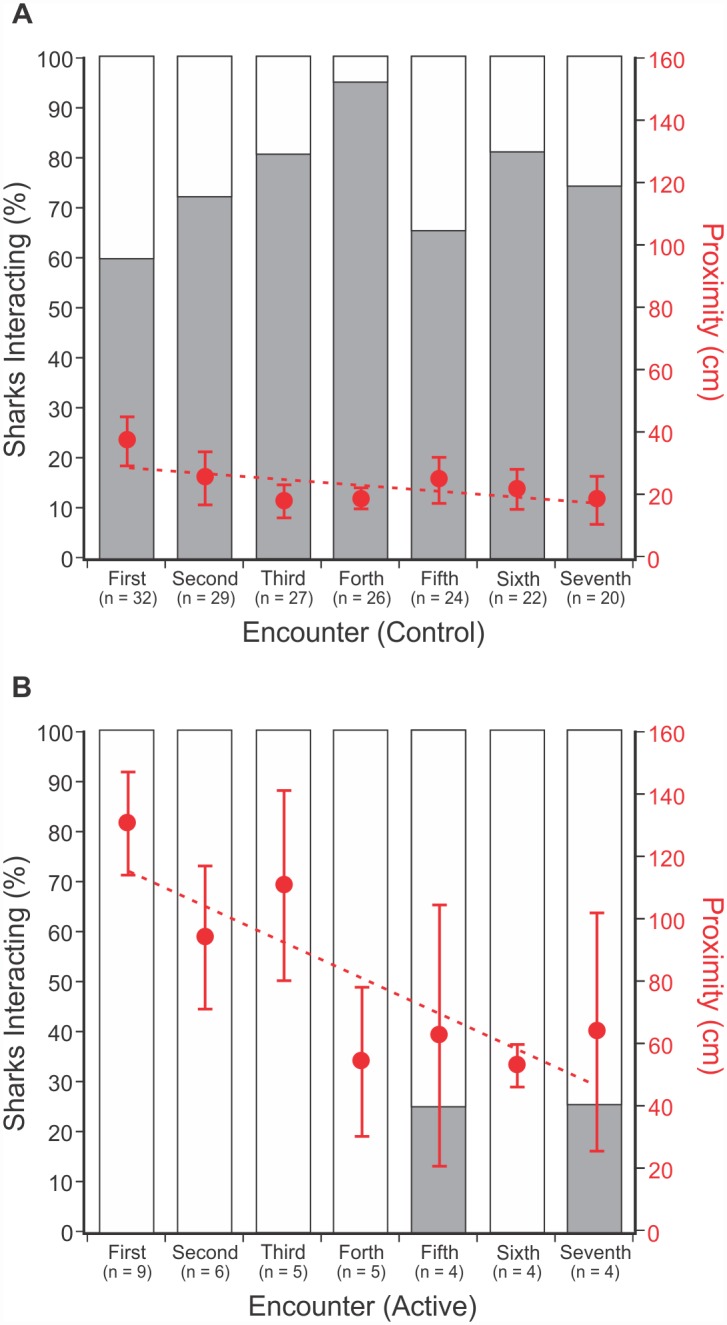
Bar graphs show the proportion of sharks that interacted (grey bar) during each encounter with a control (A) or active (B) Shark Shield^™^ treatment. Overlaid is the average proximity of sharks to the Shark Shield^™^ during each encounter (± Std. Error). Proximity trend line (Control): y = -20.876x + 323.56; Proximity trend line (Active): y = -116.37x + 1283.

Based on an individual shark’s first 7 encounters with a ReMoRA (as this is the maximum number of encounters for which there are data available in both control and active trials), when only considering interactions (not proximity), there was no evidence of habituation between encounters during control (p ≥ 0.05; Table 2: #1; Fig 6A) or active trials (p ≥ 0.05; Table 3: #1; Fig 6B). There was also no relationship observed between the proportion of sharks interacting per encounter and the total number of sharks (all p ≥ 0.05; Control: Table 2: #2; Active: Table 3: #2), or between the proportion of sharks interacting per encounter and the number of encounters (all p ≥ 0.05; Control: Table 2: #3; Active: Table 3: #3).

**Table 2:** Comparison of the behavioural response of *C*. *carcharias* between sharks, and between encounters, during control trials. Justification for the tests used is provided below.

Test #	Description	Statistical Test	Test Result	Probability
1	Proportion of sharks interacting/encounter	Logistic Regression	Z = 0.36	p = 0.716
2	Proportion of sharks interacting/encounter *vs*. No. of sharks	Pearson's correlation^a^	r = -0.353	p = 0.438
3	Proportion of sharks interacting/encounter *vs*. No. of encounters	Pearson's correlation^a^	r = 0.330	p = 0.469
4	Proximity/shark (all encounters)	One-way ANOVA^b^^,^^c^	F_28_ = 3.18	*p ≤ 0*.001*
5	Proximity/encounter (all sharks)	One-way ANOVA^b^^,^^c^	F_6_ = 2.97	*p ≤ 0*.050*
6	Proximity/shark (all encounters) *vs*. No. of encounters/shark	Pearson's correlation^a^	r = -0.1	p = 0.604
7	Proximity/encounter (all sharks) *vs*. No. of encounters	Pearson's correlation^a^	r = -0.637	p = 0.124
8	Proximity/encounter (all sharks) *vs*. No. of sharks/encounter	Pearson's correlation^a^	r = 0.637	p = 0.124

* Denotes a significant result.

Test justification:

^(a)^ Normal distribution;

^(b)^ Normal distribution and equal variance with Log10 transformation;

^(c)^ Data unpaired.

**Table 3:** Comparison of the behavioural response of *C*. *carcharias* between sharks, and between encounters, during active trials. Justification for the tests used is provided below.

Test #	Description	Statistical Test	Test Result	Probability
1	Proportion of sharks interacting/encounter	Logistic Regression	Z = 1.08	p = 0.281
2	Proportion of sharks interacting/encounter *vs*. No. of sharks	Pearson's correlation^a^	r = -0.538	p = 0.212
3	Proportion of sharks interacting/encounter *vs*. No. of encounters	Pearson's correlation^a^	r = 0.127	p = 0.632
4	Proximity/shark (all encounters)	One-way ANOVA^b^^,^^c^	F_7_ = 2.95	*p ≤ 0*.050*
5	Proximity/encounter (all sharks)	One-way ANOVA^b^^,^^c^	F_6_ = 3.06	*p ≤ 0*.050*
6	Proximity/shark (all encounters) *vs*. No. of encounters/shark	Pearson's correlation^a^	r = -0.847	*p ≤ 0*.050*
7	Proximity/encounter (all sharks) *vs*. No. of encounters	Pearson's correlation^a^	r = -0.824	*p ≤ 0*.050*
8	Proximity/encounter (all sharks) *vs*. No. of sharks/encounter	Pearson's correlation^a^	r = 0.378	p = 0.403

* Denotes a significant result.

Test justification:

^(a)^ Normal distribution;

^(b)^ Normal distribution and equal variance;

^(c)^ Data unpaired.

Based on the same 7 encounters, when considering proximity (not interactions), there was significant variation in how close individual sharks would approach during control trials (p ≤ 0.001; Table 2: #4). The average proximity between encounters also differed significantly (p ≤ 0.05; Table 2: #5; Fig 6A). Similarly, during active trials, there was significant variation in the average proximity between individual sharks (p ≤ 0.05; Table 3: #4), and, furthermore, the average proximity between encounters also differed significantly (p ≤ 0.05; Table 3: #5; Fig 6B). However, during control trials, there was no evidence that the observed variation between individuals, or between encounters, was influenced by the number of encounters (all p ≥ 0.05; Table 2: #6 and #7; Fig 6A), or by the number of sharks (p ≥ 0.05; Table 2: #8). Whereas, during active trials, there was evidence that the observed variation in proximity between individuals, and between encounters, was significantly negatively correlated with the number of encounters (all p ≤ 0.05; Table 3: #6 and #7; Fig 6B), but not by the number of sharks (p ≥ 0.05; Table 3: #8). As a result, the more times that *C*. *carcharias* encountered the active Shark Shield^™^, the closer they would approach, decreasing their proximity by an average of 11.6 cm each encounter (Fig 6B). However, despite the apparent habituation to the electric field, the sharks did not re-encounter the active Shark Shield^™^ enough within a single trial (Table 1: #5) to reduce their proximity sufficiently to result in an interaction (Table 1: #9).

### Electric Field Gradient

**Fig 7:**
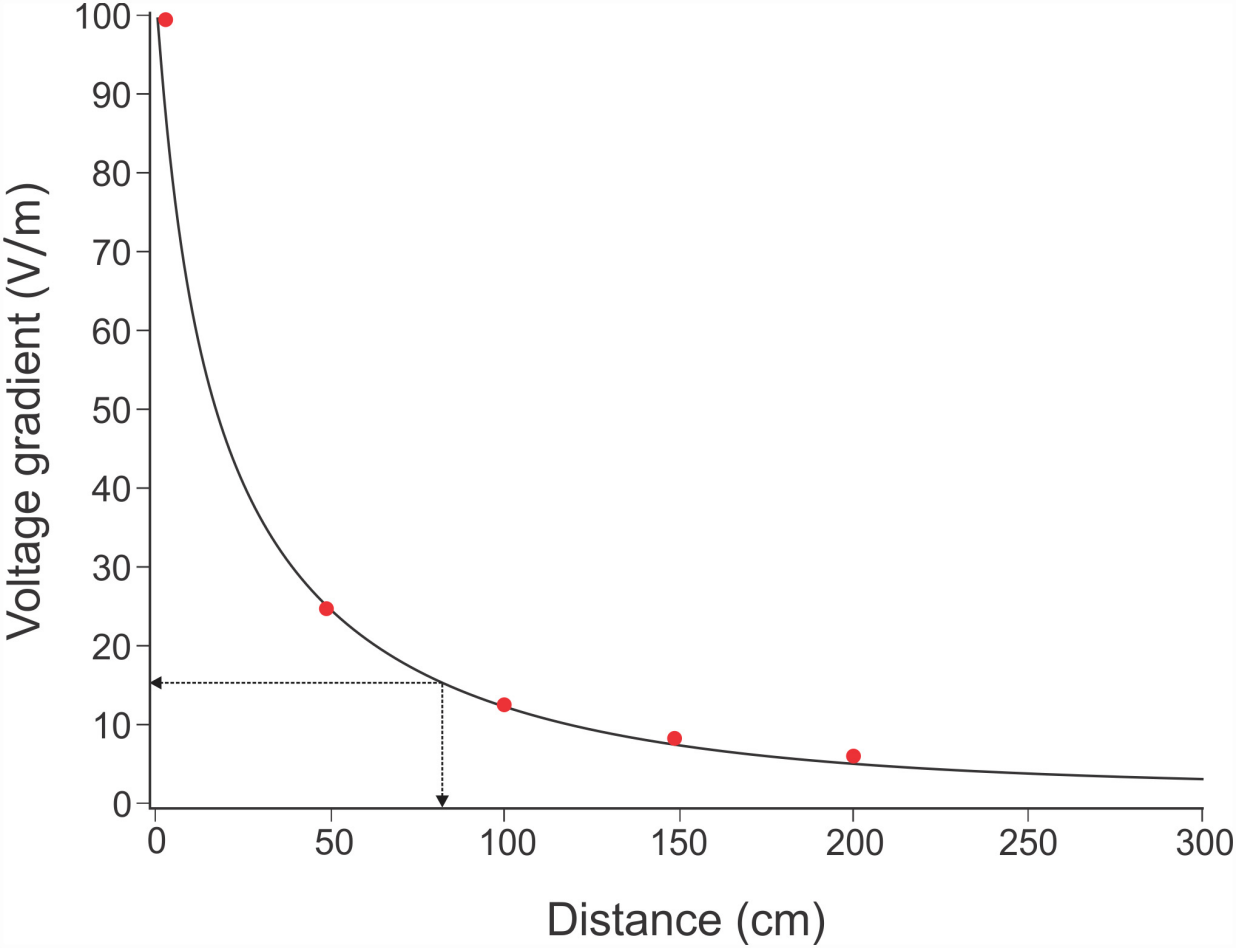
Plot to show the Shark Shield^™^ voltage gradient decline with increasing distance. The dashed arrows indicate the average proximity/encounter (82 cm) of *C*. *carcharias* and the corresponding voltage gradient (15.7 V/m); Red dots depict actual measurements recorded using the voltage gradient probe (see Fig 4). Voltage gradient curve plotted using Harris model: y = 1/(0.0101 + 0.0003x^1.1706).

Measurements of the electric field generated by the Shark Shield^™^ showed that the voltage gradient was greatest in close proximity to the electrodes of the Shark Shield^™^ and decreased rapidly with distance (Fig 7). The Shark Shield^™^ measured in this study discharged at a frequency of 1.67 Hz, with a peak voltage gradient of ≥100 V/m within 5 cm of the electrode surface (Fig 7). The gradient of the electric field at equal distances around the Shark Shield^™^ varied slightly (± 2.7%) depending on the angle of the recording probe relative to the Shark Shield’s electrodes. For consistent measurements, the gradient was plotted along the same axis, parallel to the end of the electrode. Based on the average proximity to an active Shark Shield^™^, when controlling for the effect of encounter number (82 ± 12 cm; Table 1: #14), the estimated average voltage gradient necessary to elicit a deterrent response equated to approximately 15.7 (± 2.1) V/m (Fig 7). However, as proximity has been shown to decline over subsequent encounters (Table 3: #6 and #7), the estimated voltage gradient to elicit a deterrent response during the first encounter (131 ± 10 cm) is much lower than the average and equates to approximately 9.7 (± 0.9) V/m. Therefore, based on an average decrease in proximity by 11.6 cm per encounter, the voltage tolerance of individual sharks would be expected to increase by approximately 2.6 (± 0.5) V/m per encounter.

## Discussion

In this investigation, an inactive Shark Shield^™^ (control treatment) was compared with an active Shark Shield^™^ (active treatment) to assess the effect of low voltage electric fields (<100 V/m: see Fig 7) on the behaviour of *C*. *carcharias* in Mossel Bay, when presented in close proximity to a static bait attractant. The active Shark Shield^™^ proved to be an effective deterrent at close range, with less than one-third of the number of sharks appearing in the camera’s field-of-view when compared with the control trials (Table 1: #2), and fewer sharks approaching close enough to interact with the bait canister (Table 1: #2). The active device was likely having behavioural effects beyond the field-of-view of the cameras (≥3 m away), which reduced the total number of sharks observed. Overall, there was an 83% reduction in the proportion of sharks interacting with the bait when the Shark Shield^™^ was active (Table 1: #2). These findings concur with a study by Smit and Peddemors [22] of an earlier Shark Shield^™^ model (known as the Shark POD^™^), which showed a similar reduction in shark bites on baited targets.

Huveneers et al. [21] tested the same device as in the present study (Shark Shield Freedom7^™^), but with bait located approximately 2–3 m away from the deterrent to reproduce the intended distance between the centre of the electric field produced by the deterrent and the head of a user. While a behavioural response was also observed by Huveneers et al. [21], the presence of the active Shark Shield^™^ did not have a significant effect on the proportion of times the bait was consumed by *C*. *carcharias*. In the present study, the average proximity per encounter was 82 (± 12) cm, which likely explains why Huveneers et al. [21] did not observe a significant reduction in the number of baits consumed during their testing. Combined, Huveneers et al. [21], Smit and Peddemors [22], and the present study, show that the effective deterrent range of the Shark Shield^™^ is likely <2 m, highlighting the importance of carefully considering the position of the electrodes in relation to the object or person intended to be protected by the device.

The short range effectiveness of the Shark Shield^™^ means that consideration must be given to its configuration, based on activities. For example, a swimmer or surfer using this device may find that part of their body would be outside of the protective field (Fig 5). Nevertheless, as shown by Huveneers et al. [21], a Shark Shield^™^ positioned in close proximity to a surface decoy was sufficient enough to effect the behaviour of *C*. *carcharias* and reduce surface interactions by 92.6%. The authors suggested that sharks might have been less likely to initiate an approach or breach because the electric field increased their ability to sense that the decoy was not a natural prey item. The results of Huveneers et al. [21] suggested that the behavioural response of *C*. *carcharias* to the Shark Shield^™^ was likely to be contextually-specific. In contrast to a swimmer or surfer, a scuba diver in the water column wearing a Shark Shield^™^ with a trailing antenna may be approached by a shark from any direction, which would potentially leave areas of their body unprotected. Therefore, by connecting the free end of the antenna of the Shark Shield^™^ to the scuba tank, the diver would then be completely encompassed within the protective field, which is the basic configuration of an alternative Shark Shield^™^ model, the Shark Shield Scuba 7^™^.

Speculation exists that sharks detecting an electric deterrent from a long distance away may mistake the stimulus with that of potential prey, as the reduced voltage gradient at that distance may indicate the presence of a typical prey item [57]. This has created growing concern that electric deterrents may attract sharks from a distance before repelling them at close proximity. However, no evidence was found in this study to suggest that the Shark Shield^™^ may attract sharks, which was also the view of Huveneers et al. [58]. Nevertheless, it should be noted that our field-of-view was restricted to close proximity encounters (≤3 m), and so we cannot be certain about the presence or absence of sharks beyond this area.

Once a shark was observed in the camera’s field-of-view, it would remain in the area for an average of 1 minute 42 seconds during control trials, and for an average of 58 seconds during active trials. During this time, sharks present in control trials would make an average of 8 passes within the field-of-view, and 4 passes during active trials. During control trials, most of the passes would result in an interaction with the bait canister, whereas during active trials, almost no interactions were observed (Table 1: #6; Fig 6). Despite interacting with a bait canister, sharks were never fed, as the bait attractant was sealed in a secure canister and was never removed from the ReMoRA. In control trials, sharks would repeatedly interact with the bait canister and eventually leave the area without reward. Whereas, during active trials, although sharks would repeatedly encounter the bait canister, most individuals did not interact and would also leave the area without reward. Sharks, like most animals, need to consider the energetic cost of obtaining food, and so, if a food item requires a lot of energy to obtain, it may be more efficient to seek out another, less energetically-expensive food item [59,60].

Being able to deter a shark in a single instance is important, but continuing to deter that same individual over a period of time is a key consideration in the development of an effective deterrent. No prior study of electric deterrents has provided data to support or refute their effectiveness when a shark is repeatedly exposed to the same electric field over an extended period of time. However, studies of the electrosensory system have shown that some shark species will habituate to electric stimuli, which can then affect their future behaviours with the same stimulus [31,61]. In the present study, even though *C*. *carcharias* were deterred from interacting over multiple encounters, when in the presence of an active Shark Shield^™^, they showed a degree of habituation to the electric field (Fig 6). Average proximity decreased with every encounter, possibly suggesting that sharks were becoming more tolerant of the electric field. Despite this, the Shark Shield^™^ continued to deter 89% sharks from interacting during active trials (Table 1: #2).

The average deterrent threshold for *C*. *carcharias* was calculated to be approximately 15.7 V/m (Fig 7), which is lower than the maximum voltage gradient tolerated by *S*. *lewini* (18.5 V/m) [43], but higher than the deterrent threshold suggested for *T*. *semifasciata* (9.6 V/m) [43], *C*. *obscurus* (7–10 V/m) [45], and *C*. *leucas* (3 V/m) [45]. In the case of *S*. *lewini*, Marcotte and Lowe [43] suggested that the deterrent threshold was likely to be inflated as a result of forced acclimation, as the sharks could not leave the testing area. If a shark cannot retreat from an electric stimulus, it may habituate to it over time [31], which will lessen its effectiveness as a deterrent [43]. Furthermore, as evident by the findings of Marcotte and Lowe [43], Smit and Peddemors [22], and Smith [45], behavioural responses to electric fields are likely to be species-specific, and so it is important to recognise that not all shark species may be deterred in the same way, if at all. Nevertheless, based on the electrical output of the Shark Shield^™^ (Fig 7), it is estimated that those species previously reported on would also be deterred, and their average proximity to the device (based on the voltage gradient curve shown in Fig 7) is estimated as follows: *S*. *lewini* ≥ 0.55 m, *T*. *semifasciata* ≥ 1.35 m, *C*. *obscurus* ≥ 1.28 m, and *C*. *leucas* > 2.0 m (the proximity for *T*. *semifasciata* is based on its higher reported threshold of 10 V/m, while that for *C*. *leucas* is estimated to be > 2 m as this was the limit to where the Shark Shield^™^ output was measured in this study). Although, it should be noted that given the different experimental protocols used to determine deterrent thresholds in the above named species, their behaviour around a Shark Shield^™^ is likely to vary. Therefore, the effective deterrent ranges estimated above are simply a guide based on the best available information at this time.

A number of factors will likely contribute to the effective range of an electric deterrent, including the electrosensory deterrent threshold of the species encountering it, the environmental conditions in which it is encountered, and the temperature and salinity (both of which will have a significant impact on the conductivity of the water) [62]. Other factors, such as the electric field discharge frequency, may also play an important role. The Shark Shield^™^ discharges at a frequency of 1.67Hz, which closely matches the frequency of respiratory signals produced by some teleost fishes, and elasmobranchs [57,63]. In addition, Kempster et al. [31] observed a greater behavioural response by bamboo shark embryos (*Chiloscyillium punctatum*) when the voltage gradient increased and frequencies ranged between 0.1 and 2Hz. Therefore, as voltage gradient is a limiting factor in the development of an electric deterrent, due to the potential negative effects on the users wearing them (causing involuntary muscle spasms) [64], it may be possible to increase effectiveness further by altering the frequency of the electric field discharge instead, but this remains to be tested.

The results of this study show that the Shark Shield^™^ can reduce *C*. *carcharias* interactions with a static bait (under test conditions), and provides no support to the suggestion that the Shark Shield^™^ attracts sharks. This study also provides evidence that *C*. *carcharias* show habituation to low voltage electric fields, at least over short time scales. However, despite this, sharks continued to be deterred by the Shark Shield^™^ for the duration of each trial. Although species-specific variations in deterrent threshold are likely, the fact that *C*. *carcharias* is implicated in the majority of fatal incidents globally [2] suggests that a deterrent that effectively deters this species should be an important safety consideration for a range of ocean users. Future research should compare the behavioural response of a range of shark species to similar low voltage electric fields when presented at the surface and in the water column, and also test the behavioural effect of varying the discharge frequency. This will allow predictions to be made about the appropriate use of electric deterrents for different species, and under different conditions.

## Supporting Information

S1 Table Behavioural response of *C*. *carcharias* when encountering an inactive/control (A) or active (B) Shark Shield^™^. (DOCX)

## References

[1] Muter BA, Gore ML, Gledhill KS, Lamont C, Huveneers C (2013) Australian and US news media portrayal of sharks and their conservation. Conservation Biology 27: 187–196. 10.1111/j.1523-1739.2012.01952.x 23110588

[2] West J (2011) Changing patterns of shark attacks in Australian waters. Marine and Freshwater Research 62: 744–754.

[3] Wetherbee BM, Lowe C., C G. (1994) A Review of Shark Control in Hawaii with Recommendations for Future Research. Pacific Science 4: 95–115.

[4] Reid D, Robbins W, Peddemors V (2011) Decadal trends in shark catches and effort from the New South Wales, Australia, Shark Meshing Program 1950–2010. Marine and Freshwater Research 62: 676–693.

[5] Dudley SFJ (1997) A comparison of the shark control programs of New South Wales and Queensland (Australia) and KwaZulu-Natal (South Africa). Ocean & Coastal Management 34: 1–27.

[6] House D (2014) Western Australian Shark Hazard Mitigation Drum Line Program 2014–17: Public Environmental Review. Western Australia: The Department of the Premier and Cabinet 85 p.

[7] Ferretti F, Worm B, Britten GL, Heithaus MR, Lotze HK (2010) Patterns and ecosystem consequences of shark declines in the ocean. Ecology Letters 13: 1055–1071. 10.1111/j.1461-0248.2010.01489.x 20528897

[8] Ruppert JLW, Travers MJ, Smith LL, Fortin M-J, Meekan MG (2013) Caught in the Middle: Combined Impacts of Shark Removal and Coral Loss on the Fish Communities of Coral Reefs. PLOS ONE 8: e74648 10.1371/journal.pone.0074648 24058618 PMC3776739

[9] Cliff G, Dudley SFJ (1991) Sharks caught in the protective gill nets off Natal, South Africa. 4. The bull shark Carcharhinus leucas Valenciennes. South African Journal of Marine Science 10: 253–270.

[10] Dudley SFJ, Cliff G (1993) Sharks caught in the protective gill nets off Natal, South Africa. 7. The blacktip shark Carcharhinus limbatus (Valenciennes). South African Journal of Marine Science 13: 237–254.

[11] Cliff G (1995) Sharks caught in the protective gill nets off Kwazulu-Natal, South Africa. 8. The great hammerhead shark *Sphyrna mokarran* (Ruppell). South African Journal of Marine Science 15: 105–114.

[12] Cliff G, Dudley S, Jury M (1996) Catches of white sharks in KwaZulu-Natal, South Africa and environmental influences. Great white sharks: The biology of Carcharodon carcharias: 351–362.

[13] Vianna GMS, Meekan MG, Pannell DJ, Marsh SP, Meeuwig JJ (2012) Socio-economic value and community benefits from shark-diving tourism in Palau: A sustainable use of reef shark populations. Biological Conservation 145: 267–277.

[14] Gallagher AJ, Hammerschlag N (2011) Global shark currency: the distribution, frequency, and economic value of shark ecotourism. Current Issues in Tourism 14: 797–812.

[15] Cisneros-Montemayor AM, Barnes-Mauthe M, Al-Abdulrazzak D, Navarro-Holm E, Sumaila UR (2013) Global economic value of shark ecotourism: implications for conservation. Oryx 47: 1–8.

[16] Kempster RM, Collin SP (2014) Iconic Species: Great White Sharks, Basking Sharks and Whale Sharks In: Klein EJTaN, editor. Sharks: Conservation, Governance and Management: Taylor and Francis Group pp. 352.

[17] Atwood TB, Connolly RM, Ritchie EG, Lovelock CE, Heithaus MR, Hays GC, et al (2015) Predators help protect carbon stocks in blue carbon ecosystems. Nature Clim Change advance online publication.

[18] O'Connell CP, Stroud EM, He P (2014) The emerging field of electrosensory and semiochemical shark repellents: Mechanisms of detection, overview of past studies, and future directions. Ocean & Coastal Management 97: 2–11.

[19] Hart NS, Collin SP (2015) Sharks senses and shark repellents. Integrative Zoology 10: 38–64. 10.1111/1749-4877.12095 24919643

[20] Jordan LK, Mandelman JW, McComb DM, Fordham SV, Carlson JK, Werner TB (2013) Linking sensory biology and fisheries bycatch reduction in elasmobranch fishes: a review with new directions for research. Conservation Physiology 1: 1–20. 10.1093/conphys/cot002 PMC4732448 27293586

[21] Huveneers C, Rogers PJ, Semmens JM, Beckmann C, Kock AA, Page B, et al (2013) Effects of an Electric Field on White Sharks: In Situ Testing of an Electric Deterrent. PLOS ONE 8: e62730 10.1371/journal.pone.0062730 23658766 PMC3642219

[22] Smit CE, Peddemors V (2003) Estimating the probability of a shark attack when using an electric repellent: applications. pp. 59–78.

[23] Brill R, Bushnell P, Smith L, Speaks C, Sundaram R, Stroud E, et al (2009) The repulsive and feeding-deterrent effects of electropositive metals on juvenile sandbar sharks (Carcharhinus plumbeus). Fishery Bulletin 107: 298–307.

[24] Marcotte MM, Lowe CG (2008) Behavioral responses of two species of sharks to pulsed, direct current electrical fields: Testing a potential shark deterrent. Marine Technology Society Journal 42: 53–61.

[25] Cliff G, Dudley SFJ (1992) Protection against shark attack in South Africa, 1952–90. Marine and Freshwater Research 43: 263–272.

[26] Broad A, Knott N, Turon X, Davis AR (2010) Effects of a shark repulsion device on rocky reef fishes: no shocking outcomes. Marine Ecology Progress Series 408: 295–298.

[27] Kalmijn AJ (1972) Bioelectric fields in sea water and the function of the ampullae of Lorenzini in elasmobranch fishes. SIO Reference, Scripps Institution of Oceanography, UC San Diego.

[28] Kempster RM, Egeberg CA, Hart NS, Collin SP (2015) Electrosensory-driven feeding behaviours of the Port Jackson shark (*Heterodontus portusjacksoni*) and western shovelnose ray (*Aptychotrema vincentiana*). Marine and Freshwater Research 67: 187–194.

[29] Kajiura SM, Fitzgerald TP (2009) Response of juvenile scalloped hammerhead sharks to electric stimuli. Zoology 112: 241–250. 10.1016/j.zool.2008.07.001 19097876

[30] Egeberg CA, Kempster RM, Theiss SM, Hart NS, Collin SP (2014) The distribution and abundance of electrosensory pores in two benthic sharks: a comparison of the wobbegong shark, *Orectolobus maculatus*, and the angel shark, *Squatina australis*. Marine and Freshwater Research 65: 1003–1008.

[31] Kempster RM, Hart NS, Collin SP (2013) Survival of the Stillest: Predator Avoidance in Shark Embryos. PLOS ONE 8: e52551 10.1371/journal.pone.0052551 23326342 PMC3541397

[32] Sisneros JA, Tricas TC (2002) Neuroethology and life history adaptations of the elasmobranch electric sense. Journal of Physiology-Paris 96: 379–389. 10.1016/S0928-4257(03)00016-0 14692486

[33] Kempster RM, Garza-Gisholt E, Egeberg CA, Hart NS, O’Shea OR, Collin SP (2013) Sexual dimorphism of the electrosensory system: a quantitative analysis of nerve axons in the dorsal anterior lateral line nerve of the blue spotted fantail stingray (*Taeniura lymma*). Brain, Behavior and Evolution 81: 1–10. 10.1159/000351700 23817033

[34] Kalmijn AJ (1971) The electric sense of sharks and rays. Journal of Experimental Biology 55: 371–383. 5114029 10.1242/jeb.55.2.371

[35] Kalmijn AJ (1974) The detection of electric fields from inanimate and animate sources other than electric organs In: Fessard A, editor. Handbook of senory physiology. Berlin: Springer Verlag pp. 147–200.

[36] Kalmijn AJ (1973) Electro-orientation in sharks and rays: theory and experimental evidence; Oceanography SIo, editor. United States Office of Naval Research: National Technical Information Service, US Dept. of Commerce.

[37] Kalmijn AJ, Weinger MB (1981) An electrical simulator of moving prey for the study of feeding strategies in sharks, skates, and rays. Annals of Biomedical Engineering 9: 363–367.

[38] Jordan LK, Mandelman JW, Kajiura SM (2011) Behavioral responses to weak electric fields and a lanthanide metal in two shark species. Journal of Experimental Marine Biology and Ecology 409: 345–350.

[39] Kajiura SM (2003) Electroreception in neonatal bonnethead sharks, Sphyrna tiburo. Marine Biology 143: 603–611.

[40] Kalmijn AJ (1988) Detection of weak electric fields Sensory biology of aquatic animals. pp. 151–186.

[41] Kalmijn AJ (1982) Electric and magnetic field detection in elasmobranch fishes. Science 218: 916 7134985 10.1126/science.7134985

[42] Kempster RM (2014) The role of electroreception in elasmobranchs [PhD]. Perth, WA: University of Western Australia.

[43] Marcotte MM, Lowe CG (2008) Behavioral Responses of Two Species of Sharks to Pulsed, Direct Current Electrical Fields: Testing a Potential Shark Deterrent. Marine Technology Society Journal 42: 53–61.

[44] Smith E (1991) Electric shark barrier: initial trials and prospects. Power Engineering Journal 5: 167–176.

[45] Smith E (1974) Electro-physiology of the electrical shark-repellant. The Transactions of the Institute of Electrical Engineers 65: 1–20.

[46] Ryklief R, Pistorius PA, Johnson R (2014) Spatial and seasonal patterns in sighting rate and life-history composition of the white shark Carcharodon carcharias at Mossel Bay, South Africa. African Journal of Marine Science 36: 449–453.

[47] Letessier TB, Meeuwig JJ, Gollock M, Groves L, Bouchet PJ, Chapuis L, et al (2013) Assessing pelagic fish populations: The application of demersal video techniques to the mid-water environment. Methods in Oceanography 8: 41–55.

[48] Letessier TB, Juhel J-B, Vigliola L, Meeuwig JJ (2015) Low-cost small action cameras in stereo generates accurate underwater measurements of fish. Journal of Experimental Marine Biology and Ecology 466: 120–126.

[49] Harvey E, Shortis M (1995) A system for stereo-video measurement of sub-tidal organisms. Marine Technology Society Journal 29: 10–22.

[50] Kock A, Johnson R (2006) White shark abundance: not a causative factor in numbers of shark bite incidents. Finding a balance: White shark conservation and recreational safety in the inshore waters of Cape Town, South Africa: 1–19.

[51] SeaGIS (2016) CAL—Stereo Camera Calibration. SeaGIS Pty. www.seagis.com.au.

[52] Dunbrack RL (2006) In situ measurement of fish body length using perspective-based remote stereo-video. Fisheries Research 82: 327–331.

[53] Langlois T, Harvey E, Fitzpatrick B, Meeuwig J, Shedrawi G, Watson D (2010) Cost-efficient sampling of fish assemblages: comparison of baited video stations and diver video transects. Aquatic Biology 9: 155–168.

[54] Zintzen V, Anderson MJ, Roberts CD, Harvey ES, Stewart AL, Struthers CD (2012) Diversity and Composition of Demersal Fishes along a Depth Gradient Assessed by Baited Remote Underwater Stereo-Video. PLOS ONE 7: e48522 10.1371/journal.pone.0048522 23119045 PMC3485343

[55] Watson DL, Harvey ES, Fitzpatrick BM, Langlois TJ, Shedrawi G (2010) Assessing reef fish assemblage structure: how do different stereo-video techniques compare? Marine Biology 157: 1237–1250.

[56] Watson DL, Harvey ES, Anderson MJ, Kendrick GA (2005) A comparison of temperate reef fish assemblages recorded by three underwater stereo-video techniques. Marine Biology 148: 415–425.

[57] Bedore CN, Kajiura SM (2013) Bioelectric Fields of Marine Organisms: Voltage and Frequency Contributions to Detectability by Electroreceptive Predators. Physiological and Biochemical Zoology 86: 298–311. 10.1086/669973 23629880

[58] Huveneers C, Rogers PJ, Semmens JM, Beckmann C, Kock AA, Page B, et al (2012) Effects of the Shark Shield electric deterrent on the behaviour of white sharks (Carcharodon carcharias). South Australian Research and Development Institute (SARDI).

[59] Brown JS, Kotler BP (2004) Hazardous duty pay and the foraging cost of predation. Ecology Letters 7: 999–1014.

[60] Martin RA, Hammerschlag N, Collier RS, Fallows C (2005) Predatory behaviour of white sharks (Carcharodon carcharias) at Seal Island, South Africa. Journal of the Marine Biological Association of the United Kingdom 85: 1121–1135.

[61] Kimber JA S D, Bellamy PH, Gill AB (2014) Elasmobranch cognitive ability: using electroreceptive foraging behaviour to demonstrate learning, habituation and memory in a benthic shark. Animal Cognition 17: 55–65. 10.1007/s10071-013-0637-8 23620366

[62] McGowan DW, Kajiura SM (2009) Electroreception in the euryhaline stingray, Dasyatis sabina. Journal of Experimental Biology 212: 1544–1552. 10.1242/jeb.025247 19411548

[63] Sisneros JA, Tricas TC (2002) Ontogenetic changes in the response properties of the peripheral electrosensory system in the Atlantic stingray (Dasyatis sabina). Brain Behavior and Evolution 59: 130–140. 10.1159/000064160 12119532

[64] Bikson M (2004) A review of hazards associated with exposure to low voltages. Center TGSaU, translator. New York: University of New York 18 p.

