# Supplementary material for: How Close is too Close? The Effect of a Non-Lethal Electric Shark Deterrent on White Shark Behaviour
Source: PLoS One. 2016 Jul 1;11(7):e0157717. doi: 10.1371/journal.pone.0157717 (PMC4930202; doi:10.1371/journal.pone.0157717)
Supplement: S1 Table — (DOCX) [file pone.0157717.s001.docx]

**Supplementary Table 1.** Behavioural response of *C. carcharias* when encountering an inactive/control (A) or active (B) Shark Shield^TM^.

**A**

|  |  |  |  |  |  |  | **Range** | | |
| --- | --- | --- | --- | --- | --- | --- | --- | --- | --- |
| **Test #** | **Description (Control Only)** | **N** | **(Mean** | ± | **Standard Error)** | **Median** | **Min** | **-** | **Max** |
|  |  |  |  |  |  |  |  |  |  |
| 1 | Proportion of deployments with sharks present | 22 | 0.68 | ± | 0.10 | n/a | n/a | - | n/a |
| 2 | Proportion of sharks interacting | 32 | 0.94 | ± | 0.04 | n/a | n/a | - | n/a |
| 3 | Proportion of sharks interacting (type 2 only) | 32 | 0.81 | ± | 0.07 | n/a | n/a | - | n/a |
| 4 | Proportion of sharks interacting (first encounter only) | 32 | 0.59 | ± | 0.09 | n/a | n/a | - | n/a |
|  |  |  |  |  |  |  |  |  |  |
| 5 | No. of encounters/shark | 32 | 8.03 | ± | 1.02 | 7.50 | 1.00 | - | 22.00 |
| 6 | No. of interactions/shark | 32 | 6.40 | ± | 0.84 | 5.00 | 0.00 | - | 20.00 |
|  |  |  |  |  |  |  |  |  |  |
| 7 | Arrival time of first shark on screen/trial (mins) | 15 | 43:06 | ± | 07:04 | 32:44 | 05:41 | - | 82:13 |
| 8 | Time taken to first interaction/shark (mins) | 29 | 00:11 | ± | 00:04 | 00:01 | 00:01 | - | 01:38 |
| 9 | Total time in area/shark (mins) | 32 | 01:42 | ± | 00:16 | 01:08 | 00:01 | - | 04:48 |
| 10 | Time between encounters/shark (mins) | 29 | 00:14 | ± | 00:01 | 00:13 | 00:07 | - | 00:31 |
| 11 | Time between encounters/encounter (mins) | 8 | 00:14 | ± | 00:00 | 00:13 | 00:11 | - | 00:18 |
|  |  |  |  |  |  |  |  |  |  |
| 12 | Proximity/shark (first encounter only) (cm) | 25 | 38.10 | ± | 4.90 | 28.20 | 3.70 | - | 104.2 |
| 13 | Proximity/shark (all encounters) (cm) | 29 | 26.20 | ± | 2.33 | 24.93 | 7.07 | - | 60.70 |
| 14 | Proximity/encounter (all sharks) (cm) | 7 | 24.01 | ± | 2.70 | 21.77 | 18.36 | - | 38.09 |
|  |  |  |  |  |  |  |  |  |  |

**B**

|  |  |  |  |  |  |  | **Range** | | |
| --- | --- | --- | --- | --- | --- | --- | --- | --- | --- |
| **Test #** | **Description (Active Only)** | **N** | **(Mean** | ± | **Standard Error)** | **Median** | **Min** | **-** | **Max** |
|  |  |  |  |  |  |  |  |  |  |
| 1 | Proportion of deployments with sharks present | 22 | 0.27 | ± | 0.10 | n/a | n/a | - | n/a |
| 2 | Proportion of sharks interacting | 9 | 0.11 | ± | 0.11 | n/a | n/a | - | n/a |
| 3 | Proportion of sharks interacting (type 2 only) | 9 | 0.11 | ± | 0.11 | n/a | n/a | - | n/a |
| 4 | Proportion of sharks interacting (first encounter only) | 9 | 0.00 | ± | 0.00 | n/a | n/a | - | n/a |
|  |  |  |  |  |  |  |  |  |  |
| 5 | No. of encounters/shark | 9 | 4.44 | ± | 1.04 | 4.00 | 1.00 | - | 9.00 |
| 6 | No. of interactions/shark | 9 | 0.22 | ± | 0.22 | 0.00 | 0.00 | - | 2.00 |
|  |  |  |  |  |  |  |  |  |  |
| 7 | Arrival time of first shark on screen/trial (mins) | 6 | 52:12 | ± | 17:02 | 41:06 | 13:32 | - | 83:30 |
| 8 | Time taken to first interaction/shark (mins) | 1 | 01:18 | ± | n/a | n/a | n/a | - | n/a |
| 9 | Total time in area/shark (mins) | 9 | 00:58 | ± | 00:16 | 01:31 | 00:01 | - | 01:47 |
| 10 | Time between encounters/shark (mins) | 6 | 00:19 | ± | 00:03 | 00:16 | 00:12 | - | 00:35 |
| 11 | Time between encounters/encounter (mins) | 8 | 00:16 | ± | 00:02 | 00:16 | 00:09 | - | 00:30 |
|  |  |  |  |  |  |  |  |  |  |
| 12 | Proximity/shark (first encounter only) (cm) | 6 | 131.30 | ± | 10.30 | 137.20 | 95.60 | - | 155.30 |
| 13 | Proximity/shark (all encounters) (cm) | 8 | 98.90 | ± | 14.80 | 93.70 | 38.80 | - | 155.30 |
| 14 | Proximity/encounter (all sharks) (cm) | 7 | 81.80 | ± | 11.50 | 65.10 | 54.30 | - | 131.30 |
|  |  |  |  |  |  |  |  |  |  |
